# Identification of reference genes for quantitative PCR during C3H10T1/2 chondrogenic differentiation

**DOI:** 10.1007/s11033-019-04713-x

**Published:** 2019-03-07

**Authors:** Serena Cappato, Francesca Giacopelli, Laura Tonachini, Roberto Ravazzolo, Renata Bocciardi

**Affiliations:** 1 Department of Neurosciences, Rehabilitation, Ophthalmology, Genetics, Maternal and Child Health (DINOGMI), Università degli Studi di Genova, 16132 Genova, Italy; 2 Medical Genetics Unit, IRCCS Istituto Giannina Gaslini, 16147 Genova, Italy

**Keywords:** qPCR, C3H10T1/2, Reference genes, Gene expression, Chondrogenesis, GeNorm

## Abstract

C3H10T1/2, a mouse mesenchymal stem cell line, is a well-known in vitro model of chondrogenesis that can be easily employed to recapitulate some of the mechanisms intervening in this process. Moreover, these cells can be used to validate the effect of candidate molecules identified by high throughput screening approaches applied to the development of targeted therapy for human disorders in which chondrogenic differentiation may be involved, as in conditions characterized by heterotopic endochondral bone formation. Chondrogenic differentiation of C3H10T1/2 cells can be monitored by applying quantitative polymerase chain reaction (qPCR), one of the most sensitive methods that allows detection of small dynamic changes in gene expression between samples obtained under different experimental conditions. In this work, we have used qPCR to monitor the expression of specific markers during chondrogenic differentiation of C3H10T1/2 cells in micromass cultures. Then we have applied the geNorm approach to identify the most stable reference genes suitable to get a robust normalization of the obtained expression data. Among 12 candidate reference genes (*Ap3d1, Csnk2a2, Cdc40, Fbxw2, Fbxo38, Htatsf1, Mon2, Pak1ip1, Zfp91, 18S, ActB, GAPDH*) we identified *Mon2* and *Ap3d1* as the most stable ones during chondrogenesis. *ActB, GAPDH* and *18S*, the most commonly used in the literature, resulted to have an expression level too high compared to the differentiation markers (*Sox9, Collagen type 2a1, Collagen type 10a1* and *Collagen type 1a1*), therefore are actually less recommended for these experimental conditions. In conclusion, we identified nine reference genes that can be equally used to obtain a robust normalization of the gene expression variation during the C3H10T1/2 chondrogenic differentiation.

**Electronic supplementary material:**

The online version of this article (10.1007/s11033-019-04713-x) contains supplementary material, which is available to authorized users.

## Introduction

C3H10T1/2 cells are a mouse cell line functionally similar to mesenchymal stem cells [1] that can be induced to differentiate towards chondrocyte, osteoblast and adipocyte lineages when cultured in presence of specific culture media [2–4]. In particular, these cells represent an established cellular model to simulate in vitro chondrogenesis, because they do not spontaneously differentiate under normal culture conditions, but can be induced by cellular condensation (high density micromass culture) and treatment with bone morphogenetic protein 2 (BMP2) [3, 5–8].

Due to limitations of primary cell lines and complexity of animal models, in the last decades, various in vitro cellular models with chondrogenic capabilities have been established to have cost effective, reproducible and simple experimental systems available to study the mechanisms that underlie chondrogenesis [8]. These in vitro models can be successfully applied also to recapitulate some of the crucial events that might be deregulated in pathological conditions.

Fibrodysplasia Ossificans Progressiva (FOP, MIM135100) is one of the most severe and disabling disorder due to extra-skeletal ossification (heterotopic ossification, HO). The disease is caused by mutations of the *ACVR1* gene encoding a type I receptor for bone morphogenetic proteins (BMPs), and is characterized by ectopic bone formation affecting skeletal muscles, ligaments and tendons [9, 10]. Early FOP lesions are characterized by local inflammatory response, muscle degeneration and fibro-proliferative reaction, followed by mesenchymal condensation, chondrogenesis and bone neoformation. Therefore, cartilage differentiation can be considered a crucial step in FOP pathogenesis. Regarding the origin of cells undergoing the aberrant differentiation process, the source of the progenitors involved is still actively investigated and debated, and according to recent studies is likely to be heterogeneous [11–15].

So far, no specific treatment is available to cure FOP and prevent the occurrence of the ossification flares. However, in the last years many effort have been devoted to study the cellular and molecular mechanisms altered in the disease and the advancements of the research in the field has provided targets to develop specific therapies by both drug discovery or drug repositioning approaches (for a revision see [11, 16]).

In vitro cellular models like C3H10T1/2 cells represent an essential tool to evaluate compounds identified by high throughput screening (HTS) of large collections of small molecules to find out candidate “hits” to be further developed as targeted therapy in diseases in which chondrogenesis represents a crucial step as in FOP. Indeed, primary screenings provide a list of candidate molecules that need to be validated in reliable secondary assays to confirm their effect on defined targets and to investigate pathways involved in their activity.

The progression of chondrogenic differentiation in C3H10T1/2 micromass cultures can be monitored by applying specific histological stainings such as Alcian Blue or Toluidin Blue, able to detect the deposition of extra-cellular matrix glycosaminoglycans; or by verifying the expression of differentiation markers through immunohistochemical assays with specific antibodies; by using quantitative real-time PCR (qPCR) to evaluate changes in the expression of differentiation markers at different stages of the process.

qPCR is definitely one of the most sensitive methods that allows detection of small dynamic changes in gene expression between samples, therefore it is mandatory to proceed with extreme care in every step of the process, from sample preparation to data interpretation.

MIQE (minimum information for publication of quantitative real-time PCR experiments) guidelines help to define the most important steps from RNA to qPCR to minimize errors and the minimum set of information required to evaluate the reliability of qPCR data [17]. According to these guidelines, after accurate evaluation of RNA quality and cDNA synthesis, it is essential to rely on the use of solid reference genes. Moreover, it is highly recommended to use at least two different reference genes in order to normalize the expression of mRNA under analysis.

Nevertheless, few studies report a systematic screening of panels of reference genes before the choice of the best candidates to be used in relationship to the cell type, differentiation stage and experimental conditions [18]. Very frequently, only a single gene is applied to normalize, and the most commonly used remain Glyceraldehyde 3-phosphate dehydrogenase (*GAPDH*), β-actin (*ActB*) and *18S* ribosomal RNA also in C3H10T1/2 differentiation studies [19–21].

Reference genes stability can be evaluated by several methods. The most popular rely on the use of dedicated programs such as geNorm [22], BestKeeper [23], and NormFinder [24] that are based on different algorithms, but all evaluate the variance in Cq values (quantification cycle, the cycle at which fluorescence from amplification exceeds the background fluorescence) of candidate reference genes across different samples, cell types, experimental conditions, etc.

In the current work, PrimerDesign geNorm^PLUS^ kit was employed to screen a panel of 12 reference genes in order to select the best candidates to be applied in monitoring the chondrogenic differentiation process in C3H10T1/2 cells.

To this aim, C3H10T1/2 were cultured in the appropriate conditions to induce chondrogenesis and used to select the best reference genes to obtain a reliable expression profile of differentiation markers which could be applied in different experimental settings.

## Materials and methods

### Cell culture

C3H10T1/2 cells were purchased from ATCC cell biology collection (C3H/10T1/2, Clone 8, ATCC® CCL-226™). Cells were used between the 5th and 15th passage and were routinely cultured in complete medium consisting of MEM with Earle’s Salts (Euroclone® S.p.a) supplemented with 10% fetal bovine serum (FBS, Gibco, ThermoFisher Scientific), 2 mM Glutamine, 100 U/ml Penicillin, 0.1 mg/ml Streptomycin (Euroclone® S.p.a). Cells were cultured at 37 °C in a humidified atmosphere with 5% CO_2_.

### C3H10T1/2 and chondrocyte-like differentiation

In order to induce chondrocyte-like differentiation, micromass cultures were obtained as described by Denker et al., 1999 [3]. Briefly, cells were trypsinized and 10^7^ cells/ml were resuspended in Ham’s F12 medium with 10% fetal bovine serum. 10 µl drop of cell suspension was placed in the center of a well in a standard 24-well plate (Euroclone® S.p.a). The cells were allowed to adhere for 2–3 h at 37 °C and 5% CO_2_, and then 500 µl of medium containing 100 ng/ml of BMP2 (Peprotech) was added to each well. Medium was replaced every 3 days.

To check chondrocyte differentiation, cells were either processed for RNA extraction or for Alcian Blue staining to verify glycosaminoglycans deposition. To this aim, micromass cultures were rinsed with Dulbecco’s phosphate-buffered saline (DPBS without Ca and Mg, Euroclone® S.p.a), fixed for 10 min in 4% Paraformaldehyde solution (Santa Cruz Biotechnology, Inc.) and stained overnight with a mixture containing 0.05% w/v Alcian Blue 8GX (Sigma-Aldrich), 0.2 M sodium acetate buffer, pH5.8 and 0.5 M MgCl_2_.

### Quantitative PCR primers

In this study, we used the Mouse geNorm^PLUS^ kit (PrimerDesign) that includes a list of 12 reference genes (*Ap3d1, Csnk2a2, Cdc40, Fbxw2, Fbxo38, Htatsf1, Mon2, Pak1ip1, Zfp91, 18S, ActB, GAPDH*) selected for their high stability level in different biological samples and treatment conditions. Oligonucleotides detect all the transcript variants and are PrimerDesign proprietary information (See Table S1 for accession numbers and anchor nucleotide of the assays). Primers sequences of the other genes used in this work are shown below and were selected from the literature [25, 26] to produce mouse-specific amplicons. Primer sequences: *Sox9*, Fwd GAGCCCGATCTGAAGAAGGA, Rev GCTTGACGTGCGGCTTGTTC (151 bp, [25]); Collagen type 2a1, (*Col2a1*) Fwd CACCAAATTCCTGTTCAGCC, Rev TGCACGAAACACACTGGTAAG (124 bp, [25]); Collagen type 10a1, (*Col10a1*) Fwd TTCTGCTGCTAATGTTCTTGACC, Rev GGGATGAAGTATTGTGTCTTGGG (115 bp, [26]); Collagen type 1a1, (*Col1a1*) Fwd ATGCCGCGACCTCAAGATG, Rev TGAGGCACAGACGGCTGAGTA (153 bp, [26]).

### RNA extraction and cDNA synthesis

Total RNA was isolated from T0, T1, T3, T6 and T13 time points by using the RNeasy Plus Mini kit (Qiagen) according to the manufacturers' protocol. RNA quality and concentration were established by electrophoresis on 1% Agarose gel (Fig S1) and by nanodrop spectrophotometer (ThermoFisher Scientific). All RNA samples displayed 260/280 ratios > 2.0 and first strand cDNA was obtained from 1 µg RNA, using the iScript reverse transcription supermix for RT-qPCR (Bio-Rad), according to the manufacturers’ instructions. cDNA preparations were diluted six-fold prior to quantitative PCR analysis.

### Quantitative PCR (qPCR)

Gene expression profiles were evaluated through qPCR using the SYBER Green system, (IQ™ SYBER® Green Supermix, Bio-Rad). Reactions were prepared in duplicate in 20 µl volumes using 5 µl of diluted cDNA (around 25 ng cDNA assuming 1:1 synthesis) per well. qPCR was performed on the IQ5 instrument from Bio-Rad with the following protocol: 95 °C for 3 min, 40 amplification cycles: denaturation at 95 °C for 30 s, annealing at 60 °C for 30 s and extension at 72 °C for 40 s. The presence of single specific amplification product was checked by melting curve analysis and template-free controls in all runs performed. All samples were quantified in the same run for a given reference gene and reaction efficiencies were estimated by standard dilution curve and analysis of individual traces.

Quantification cycle (Cq) values for each replicate reaction were extracted from IQ5 analysis and analyzed through the qBase^PLUS^ software (Biogazelle, https://www.qbaseplus.com/) to calculate geNorm values. Details about geNorm algorithm are described in Vandesompele et al., 2002 [22]. Relative expression of differentiation markers was calculated by the ∆Ct Method, derived as a modification of the 2^−∆∆Ct^ (Livak) approach [27]. The applied ∆Ct Method uses the difference between reference and target Ct values for each sample, (Ratio (reference/target) = 2 ^Ct(reference)−Ct(target)^).

### Statistical analysis

The results obtained from reference genes study were analyzed by the qBase^PLUS^ software (Biogazelle). Experiments to evaluate gene expression profile by qPCR were performed from three independent biological replicates and the results were represented as average ± Standard Deviation of gene relative expression. The unpaired two-tailed *Student’s t-test* (GraphPad, https://www.graphpad.com/quickcalcs/ttest1) was applied to verify statistical significance of the observed variation. Significant variations were defined as *P < 0.05.

## Results

### Chondrogenic differentiation of C3H10T1/2

**Fig. 1:**
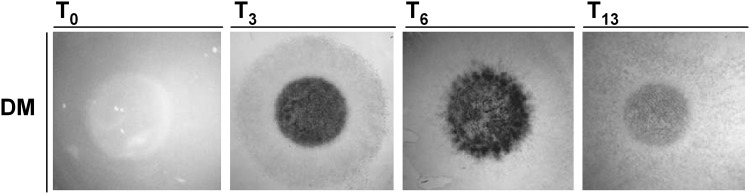
Chondrogenic differentiation of C3H10T1/2 cells. Representative C3H10T1/2 micromass cultures after 1, 3, 6 and 13 days of BMP2 treatment. Cells were fixed and stained with Alcian blue. DM, differentiation medium

C3H10T1/2 micromass cell cultures were induced to differentiate with BMP2, starting from day 1 to day 13. In order to evaluate the differentiation process, micromass cultures were stained with Alcian Blue, specific for the deposition of the matrix glycosaminoglycans, after 1, 3, 6, and 13 days of culture in inductive medium (Fig. 1). The expression level of markers associated with chondro-differentiation and cartilage formation (*Sox9, Col2a1, Col10a1* and *Col1a1*) was also monitored (Fig. S2).

### Study of 12 candidate reference genes

In parallel, cells cultured in the same conditions and blocked at the different time points were used to identify the best reference genes suitable to study gene expression variation during differentiation of C3H10T1/2. RNA quality and concentration (Fig. S1) were checked for all the samples deriving from two different groups of experiments and the expression of a panel of 12 reference genes was evaluated. qPCR results were processed with the IQ5 software and Cq values were exported to be analysed with qbase^+^ software (Table S2). Amplification efficiency, calculated from standard curves was between 1.9 and 2.10 for all genes, corresponding to 90–110% which represents the recommended range (Table S1).

**Fig. 2:**
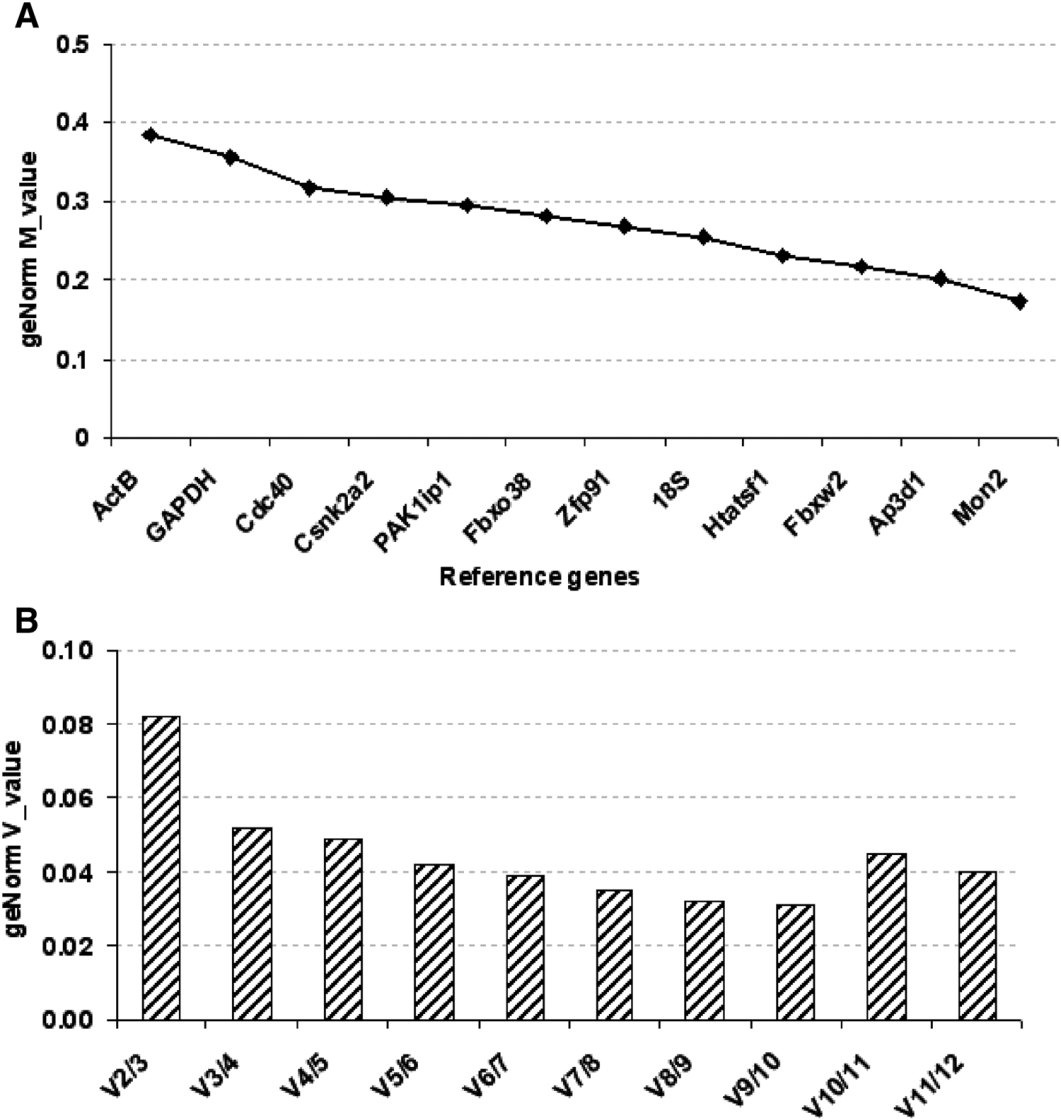
GeNorm analysis of 12 candidate reference genes. **a** geNorm M value indicates reference genes stability. The genes with geNorm M value below 0.5 can be considered for the normalization and the lower is the value, the more stable are the genes. **b** GeNorm V value represents the pairwise variation (n/n + 1) and indicates the minimum number of reference genes required for normalization. Values below 0.15 indicate the optimal number (n) of reference genes to be considered. In this study, a combination of two genes was sufficient for a correct normalization, V_2/3_ = 0.082

GeNorm study was applied following the instruction of the software. GeNorm M value represents reference gene stability and according to the geNorm manual, candidate genes with lower M value have to be considered more stable. The graph starts with the least stable gene on the left side and ends with the most stable gene on the right part, with a threshold of 0.5 between stable and unstable genes. Therefore, all the tested reference genes appeared to be stable upon the different conditions and thus suitable for gene expression analysis at different time points of C3H10T1/2 chondrogenic differentiation (Fig. 2a). However, the best candidate reference genes identified were *Mon2* and *Ap3d1*, whereas the less stable were *ActB* and *GAPDH*, curiously among the most commonly used in the literature. The geNorm V value suggests the minimum number of reference genes required for normalization. The threshold value is 0.15, below which the inclusion of an additional control is not required [22]. As shown in Fig. 2b, all the reference genes combination has V_n/n+1_ < 0.15 (n = optimal number of reference genes), suggesting that a combination of two reference genes was sufficient for a correct normalization.

### Expression profile of the differentiation markers normalized against the different reference genes

To validate geNorm analysis and reference genes selection, C3H10T1/2 micromass cultures were harvested at time 0 and after 6 days of chondrogenic differentiation and processed for qPCR assay.

**Fig. 3:**
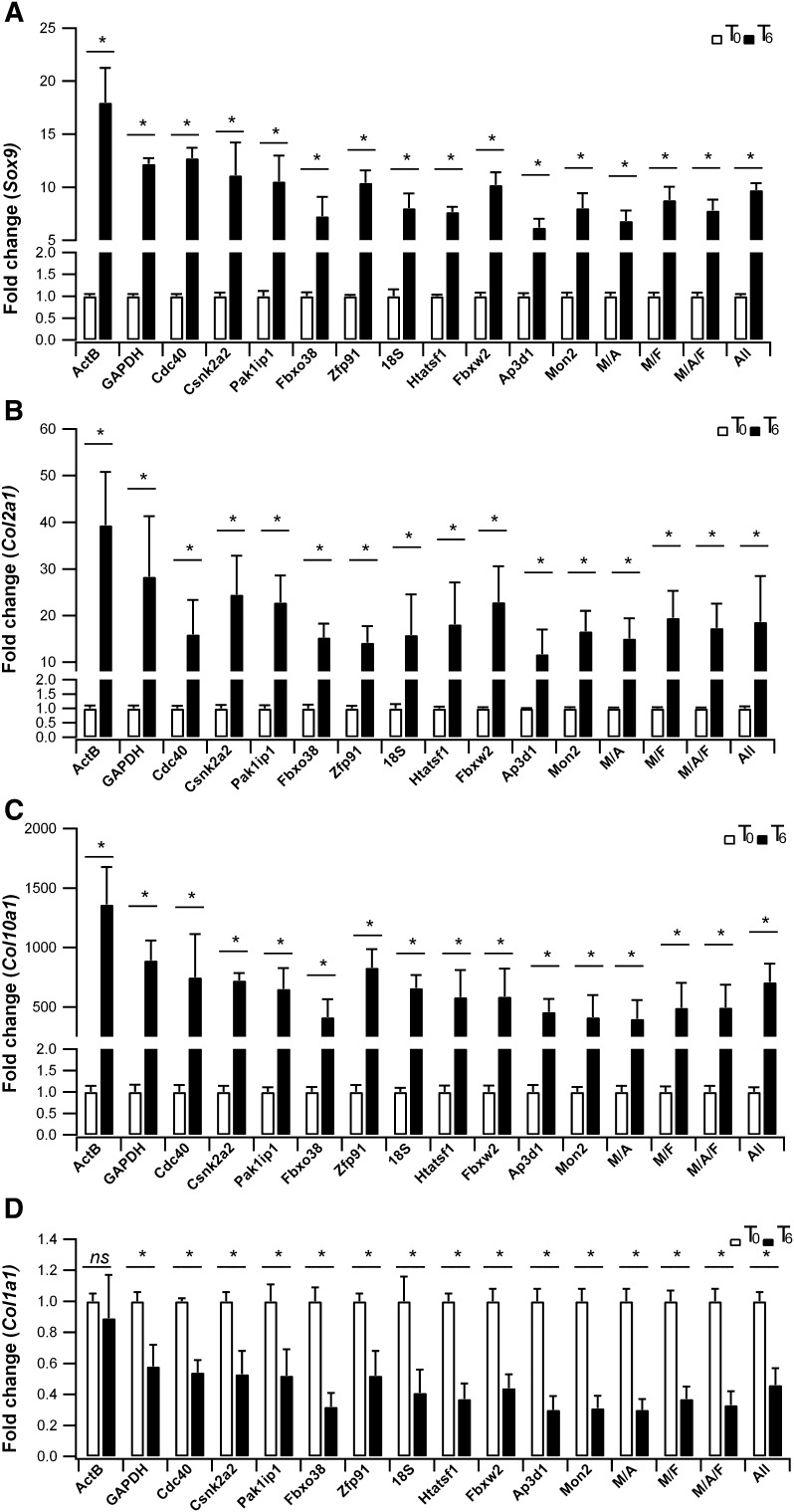
Fold-change expression of chondrogenic differentiation markers, normalized against different reference genes after 6 days of differentiation. **a***Sox9*, **b***Col2a1*, **c***Col10a1*, **d***Col1a1*. Relative expression was calculated by ∆Ct Method and histograms represent average ± Standard Deviation of three independent biological replicates. The statistical significance was evaluated by *Student’s T-test* analysis with *P < 0.05

The expression level of markers associated with chondro-differentiation and cartilage formation (*Sox9, Col2a1, Col10a1*) was obtained by the ∆Ct Method. In this evaluation, various reference genes were employed, including all single candidate reference genes, a combination of the most stable ones identified in this study, calculated by the geometric average of *Mon2&Ap3d1, Ap3d1&Fbxw2, Mon2&Ap3d1&Fbxw2* (indicated as M&A, A&F, M&A&F), and the geometric average of all 12 reference genes Ct values, (designed as ‘All’). As shown in Fig. 3 up-regulation of the chondrogenic genes *Sox9, Col2a1, Col10a1* was demonstrated by all normalization strategies. As a control, we evaluated also the expression of *Col1a1*, a gene not associated with cartilage differentiation and expected to be downregulated in micromass cultures induced by BMP2 [28]. As shown in Fig. 3, the decrease in *Col1a1* expression upon differentiating conditions does not appear significant when *ActB* is applied as normalizing gene, compared to ‘All’ and to other single or combined reference genes.

### Comparison of relative expression of 12 candidate reference genes

In order to select the proper reference genes with transcription level similar to that of target genes, Ct values deriving from qPCR assay on RNA samples at T0 and T6, were normalized against *Fbxo38*, which showed the lowest expression level.

**Fig. 4:**
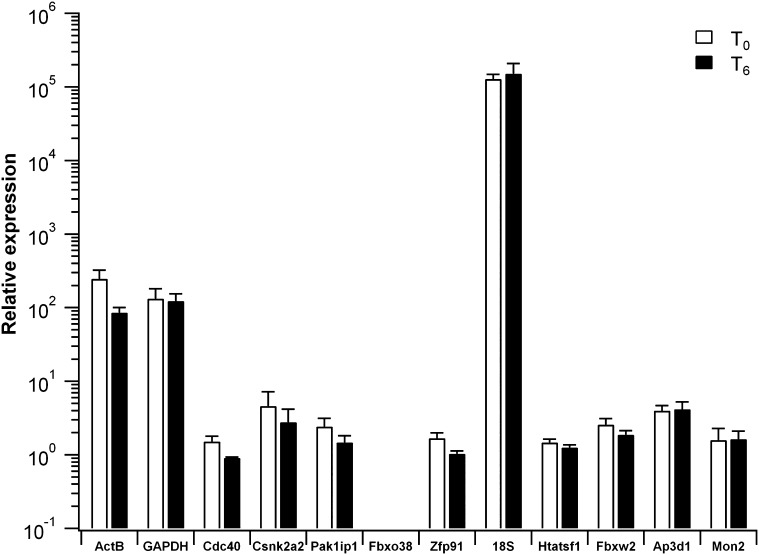
Relative expression of the 12 reference genes. Cells were analysed after 6 days of differentiation. Gene expression values were calculated using ∆Ct Method against *Fbxo38*, that showed the lowest expression. Histograms represent average and standard deviation of three independent biological replicates. Y axis was on a log_10_ scale

As reported in Fig. 4, relative expression of most of the reference genes have similar magnitude, whereas *ActB, GAPDH* and *18S* showed the highest expression in C3H10T1/2 cells.

This analysis suggested the exclusion of *ActB, GAPDH* and *18S* because their transcription levels were too far from that of target genes and this might affect the accuracy of the normalization process.

## Discussion

qPCR is one of the most sensitive method to evaluate the expression level of genes of interest under different experimental conditions, and thus in order to obtain reliable results it is crucial to select the suitable reference genes [26, 29–34].

In the last years, several algorithms were developed to identify the optimal reference genes in different cell types/tissues or treatment conditions. GeNorm [22], BestKeeper [23], and NormFinder [24] are the most frequently applied to this purpose and are reported to usually show similar results [35, 36].

To the aim of using C3H10T1/2 cells as an in vitro model of chondro-like differentiation [3], we applied the geNorm software to identify the most stable references genes suitable for normalization during the in vitro differentiation process of these cells. GeNorm has been developed according to the normalization strategy described by Vandesompele et al., 2002 [22], conceived to identify the most stably expressed control genes in a given set of tissues, and to determine the minimum number of genes required to calculate a reliable normalization factor.

In our study, we used a commercial kit of 12 murine ‘house-keeping’ genes, specifically designed by PrimerDesign for geNorm studies, and we performed two biological replicates of C3H10T1/2 chondrogenic differentiation after 1, 3, 6, and 13 days.

The first parameter generated by the geNorm^PLUS^ analysis is geNorm M, which evaluates expression stability of candidate reference genes between samples. M value represents the average pairwise variation of a particular gene with all the other control genes: the lowest M value indicates the most stable gene expression. The cut-off value is 0.5 and all the genes we analyzed in this study could be considered potential reference genes.

According to the MIQE guidelines, an accurate normalization process relies on the use of two or more reference genes and geNorm V value (pairwise variation, V_(n/n+1)_ coefficient) helps to determine the number of them required for optimal normalization. The suitable number of reference genes “n” is found when V_(n/n+1)_ drops below 0.15.

As shown in Fig. 2, V_2/3_ was less than 0.15 (V_2/3_ = 0.082) and two reference genes were sufficient for a correct normalization, ideally *Mon2* and *Ap3d1*.

To validate geNorm analysis, we evaluated the expression levels of markers specific for chondrogenic differentiation of C3H10T1/2 cells at T0 and after 6 days of micromass culture in inductive medium containing BMP2. *Sox9, Col2a1* and *Col10a1* showed high expression level after BMP2 treatment and all normalization strategies showed comparable expression level. In particular, the combination of *Mon2* and *Ap3d1, Ap3d1* and *Fbxw2*, and *Mon2, Ap3d1* and *Fbxw2*, gave similar results to the normalization obtained with ‘All’, which is considered as the most accurate strategy.

Furthermore, it is known that for accurate gene expression studies the expression level of reference genes should be similar to that of target genes [26, 37]. Therefore, we compared the expression profile of all the 12 reference genes in our cell model. Our analysis suggested that *ActB, GAPDH* and especially *18S* genes should not be considered for normalization because their expression level significantly diverges from that of the selected differentiation markers in C3H10T1/2 cells.

Moreover, the use of *ActB* as reference gene failed to efficiently detect the expected down-regulation of *Col1a1* expression in differentiating conditions [28].

This result suggested the exclusion of *ActB*, one of the most applied in expression studies, from the best reference genes group, because during the evaluation of treatment effects, also small gene expression differences may become relevant. All the other reference genes and combination analysed in this work, showed similar expression levels and pass our validation.

## Conclusions

qPCR is a potent method to study gene expression profiles in different experimental settings. Reliability of results is strongly dependent on the normalization procedure; reference genes have to be carefully selected for every tissue/cell types and for the different experimental conditions. We applied the geNorm analysis to select the best reference genes in a widely used in vitro model of chondrogenesis. According to our analysis, the most commonly used reference genes, such as *ActB, GAPDH* and *18S*, are not actually the best candidates for normalization. We suggest to use a different combination of at least two genes selected from the panel we tested, such as *Mon2* and *Ap3d1* when normalizing gene expression profiles during chondrogenesis in C3H10T1/2 cells.

## Electronic supplementary material

Below is the link to the electronic supplementary material.

Supplementary material 1—Agarose gel electrophoresis of representative RNA samples deriving from C3H10T1/2 micromasses. Integrity of RNA was represented by the presence of two bands corresponding to 28S and 18S ribosomal subunits. RNA from two different experiments for T0 (Lanes 1 and 2), and from two different experiments for T6 time point (Lanes 3 and 4) are shown (TIF 1228 KB)

Supplementary material 2—Expression level of the indicated markers after 1, 3, 6 and 13 days of chondrogenic differentiation. (A) *Sox9*, (B) *Col2a1*, (C) *Col10a1*, (D) *Col1a1*. The expression of each gene has been normalized against the geometric average of all the 12 Reference genes. Histograms represent the average ± Standard Deviation of two independent experiments (TIF 37 KB)

Supplementary material 3 (DOC 216 KB)

Supplementary material 4 (DOC 73 KB)
