# Supplementary figures and images for: Identification of reference genes for quantitative PCR during C3H10T1/2 chondrogenic differentiation
Source: Mol Biol Rep. 2019 Mar 7;46(3):3477–85. doi: 10.1007/s11033-019-04713-x (PMC6548758; doi:10.1007/s11033-019-04713-x)

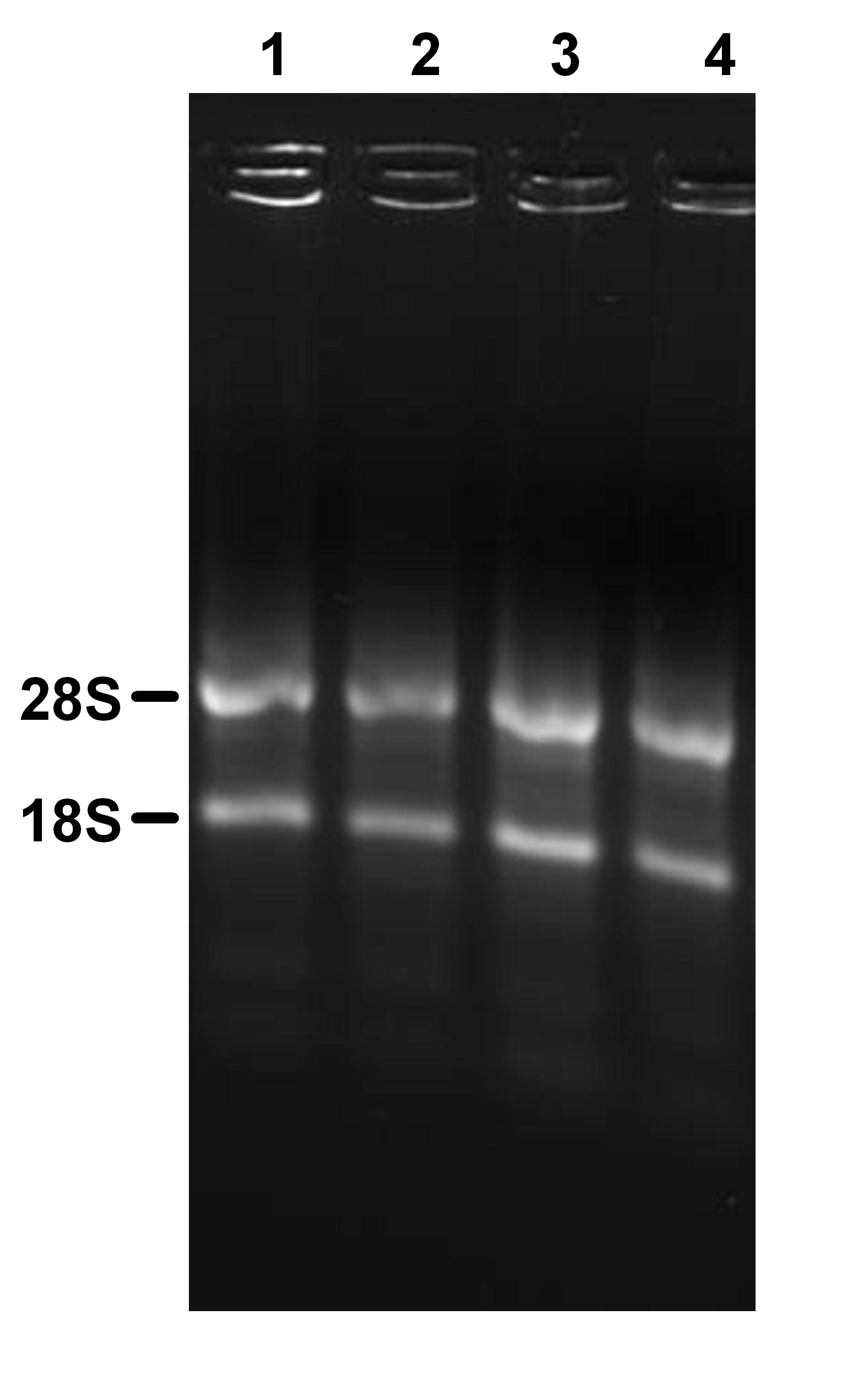

Supplement: Supplementary file 1 — Supplementary material 1—Agarose gel electrophoresis of representative RNA samples deriving from C3H10T1/2 micromasses. Integrity of RNA was represented by the presence of two bands corresponding to 28S and 18S ribosomal subunits. RNA from two different experiments for T0 (Lanes 1 and 2), and from two different experiments for T6 time point (Lanes 3 and 4) are shown (TIF 1228 KB) [file 11033_2019_4713_MOESM1_ESM.tif]

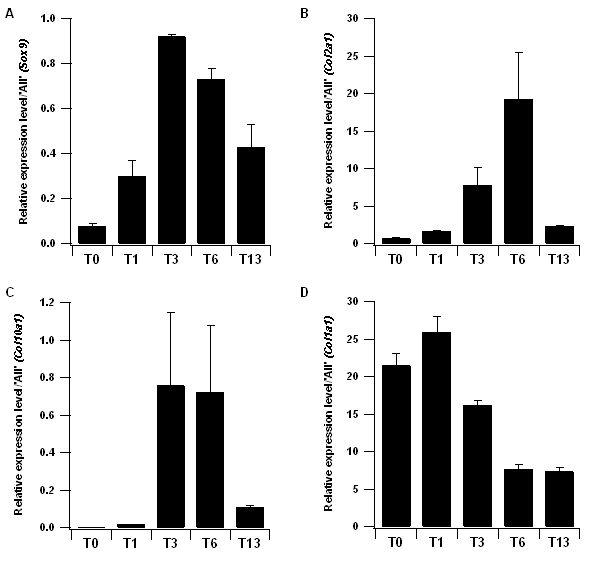

Supplement: Supplementary file 2 — Supplementary material 2—Expression level of the indicated markers after 1, 3, 6 and 13 days of chondrogenic differentiation. (A) Sox9, (B) Col2a1, (C) Col10a1, (D) Col1a1. The expression of each gene has been normalized against the geometric average of all the 12 Reference genes. Histograms represent the average ± Standard Deviation of two independent experiments (TIF 37 KB) [file 11033_2019_4713_MOESM2_ESM.tif]
