# Supplementary material for: Identification of reference genes for quantitative PCR during C3H10T1/2 chondrogenic differentiation
Source: Mol Biol Rep. 2019 Mar 7;46(3):3477–85. doi: 10.1007/s11033-019-04713-x (PMC6548758; doi:10.1007/s11033-019-04713-x)
Supplement: Supplementary file 3 — Supplementary material 3 (DOC 216 KB) [file 11033_2019_4713_MOESM3_ESM.doc]

**Table S1.** Accession number and anchor nucleotide of the assay. Amplification efficiency (E) ± Standard Error (SE) and R^2^ for 12 candidate reference genes.

|  | **Accession Number** | **Anchor Nucleotide** | **E** | **E (SE)** | **R^2^** |
| --- | --- | --- | --- | --- | --- |
| ***18S*** | NR_003278.3 | 134 | 2.01 | 0.05 | 0.99 |
| ***ActB*** | NM_007393.3 | 597 | 1.99 | 0.03 | 1.00 |
| ***Ap3d1*** | NM_007460 | 2694 | 2.07 | 0.05 | 1.00 |
| ***Cdc40*** | NM_027879 | 488 | 2.08 | 0.06 | 1.00 |
| ***Csnk2a2*** | NM_009974 | 1086 | 2.04 | 0.04 | 1.00 |
| ***Fbxo38*** | NM_134136 | 1797 | 2.01 | 0.06 | 0.99 |
| ***Fbxw2*** | NM_013890 | 655 | 2.05 | 0.05 | 1.00 |
| ***GAPDH*** | NM_008084.2 | 793 | 2.10 | 0.03 | 1.00 |
| ***Htatsf1*** | NM_028242 | 1259 | 2.04 | 0.08 | 1.00 |
| ***Mon2*** | NM_001163024 | 4707 | 2.03 | 0.03 | 1.00 |
| ***Pak1ip1*** | NM_026550 | 737 | 1.98 | 0.04 | 1.00 |
| ***Zfp91*** | NM_053009 | 1415 | 1.89 | 0.02 | 1.00 |
