# Supplementary material for: Identification of reference genes for quantitative PCR during C3H10T1/2 chondrogenic differentiation
Source: Mol Biol Rep. 2019 Mar 7;46(3):3477–85. doi: 10.1007/s11033-019-04713-x (PMC6548758; doi:10.1007/s11033-019-04713-x)
Supplement: Supplementary file 4 — Supplementary material 4 (DOC 73 KB) [file 11033_2019_4713_MOESM4_ESM.doc]

**Table S2.** Raw Ct values for all the tested reference genes in two independent differentiation experiments and for each time-point (columns 1 to 12). In the last four columns, the geometric mean of the best combination or of 'all' tested genes.

|  | ***Ap3d1*** | ***Csnk2a2*** | ***Cdc40*** | ***Fbxw2*** | ***Fbxo38*** | ***Htatsf1*** | ***Mon2*** | ***Pak1ip1*** | ***Zfp91*** | ***18S*** | ***Actb*** | ***GAPDH*** | **M&A** | **M&F** | **M&A&F** | **All** |
| --- | --- | --- | --- | --- | --- | --- | --- | --- | --- | --- | --- | --- | --- | --- | --- | --- |
| **T0** | 21.63 | 21.13 | 23.08 | 22.31 | 24.07 | 23.48 | 22.64 | 23.28 | 23.16 | 6.82 | 15.98 | 16.41 | 22.13 | 22.47 | 22.19 | 19.43 |
| **T0** | 21.84 | 20.88 | 23.12 | 22.32 | 23.71 | 23.51 | 22.72 | 22.90 | 23.20 | 7.19 | 15.82 | 16.41 | 22.28 | 22.52 | 22.29 | 19.46 |
| **T1** | 21.92 | 21.83 | 23.70 | 22.74 | 24.24 | 23.63 | 22.94 | 23.99 | 23.97 | 7.96 | 16.67 | 16.70 | 22.42 | 22.83 | 22.53 | 20.09 |
| **T1** | 21.75 | 21.90 | 23.65 | 22.58 | 24.38 | 23.36 | 22.83 | 24.25 | 23.85 | 7.85 | 16.74 | 16.67 | 22.28 | 22.70 | 22.38 | 20.04 |
| **T3** | 21.73 | 21.62 | 23.60 | 22.74 | 23.82 | 23.22 | 22.88 | 23.50 | 23.31 | 7.51 | 16.88 | 16.78 | 22.30 | 22.81 | 22.44 | 19.84 |
| **T3** | 21.73 | 21.61 | 23.59 | 22.57 | 23.81 | 23.37 | 22.84 | 23.46 | 23.38 | 7.67 | 16.84 | 16.78 | 22.28 | 22.71 | 22.38 | 19.87 |
| **T6** | 21.75 | 21.73 | 24.65 | 23.09 | 23.83 | 24.18 | 22.91 | 23.70 | 24.39 | 7.89 | 17.28 | 17.72 | 22.32 | 23.00 | 22.57 | 20.32 |
| **T6** | 21.71 | 21.79 | 24.28 | 22.99 | 23.93 | 23.98 | 22.89 | 23.77 | 24.21 | 7.90 | 17.42 | 17.45 | 22.29 | 22.94 | 22.52 | 20.27 |
| **T13** | 21.20 | 21.78 | 23.86 | 22.51 | 23.67 | 23.50 | 22.41 | 23.76 | 23.78 | 7.48 | 17.49 | 17.66 | 21.80 | 22.46 | 22.03 | 19.97 |
| **T13** | 21.18 | 21.70 | 23.64 | 22.47 | 23.63 | 23.38 | 22.46 | 23.69 | 23.72 | 7.44 | 17.31 | 17.61 | 21.81 | 22.46 | 22.03 | 19.90 |
| **T0 b** | 21.42 | 20.92 | 22.94 | 21.96 | 23.88 | 23.24 | 22.45 | 22.93 | 23.02 | 7.23 | 15.76 | 16.25 | 21.93 | 22.21 | 21.94 | 19.35 |
| **T0 b** | 21.64 | 20.84 | 22.81 | 22.22 | 23.77 | 23.24 | 22.70 | 23.16 | 22.87 | 7.44 | 15.68 | 16.09 | 22.17 | 22.46 | 22.19 | 19.41 |
| **T1 b** | 21.34 | 21.60 | 23.43 | 22.41 | 23.84 | 23.31 | 22.60 | 23.86 | 23.81 | 7.44 | 16.00 | 16.44 | 21.96 | 22.50 | 22.11 | 19.68 |
| **T1 b** | 21.49 | 21.42 | 23.27 | 22.49 | 23.70 | 23.19 | 22.66 | 23.72 | 23.66 | 7.52 | 15.97 | 16.32 | 22.07 | 22.57 | 22.21 | 19.64 |
| **T3 b** | 21.24 | 21.50 | 22.87 | 22.19 | 23.70 | 22.87 | 22.45 | 23.41 | 23.04 | 7.23 | 16.50 | 16.67 | 21.83 | 22.32 | 21.95 | 19.50 |
| **T3 b** | 20.98 | 21.47 | 22.88 | 22.04 | 23.64 | 22.75 | 22.23 | 23.38 | 22.81 | 6.84 | 16.46 | 16.56 | 21.60 | 22.14 | 21.74 | 19.32 |
| **T6 b** | 21.03 | 21.60 | 23.45 | 22.48 | 23.68 | 23.04 | 22.51 | 23.50 | 23.15 | 6.74 | 16.95 | 16.72 | 21.76 | 22.49 | 22.00 | 19.52 |
| **T6 b** | 21.02 | 21.59 | 23.28 | 22.36 | 23.77 | 22.86 | 22.43 | 23.55 | 22.87 | 6.59 | 16.97 | 16.69 | 21.72 | 22.40 | 21.93 | 19.43 |
| **T13 b** | 21.62 | 21.72 | 23.63 | 22.80 | 23.41 | 23.44 | 22.87 | 23.71 | 23.37 | 7.54 | 17.22 | 17.66 | 22.23 | 22.83 | 22.42 | 19.97 |
| **T13 b** | 21.44 | 21.74 | 23.71 | 22.65 | 23.64 | 23.44 | 22.81 | 23.83 | 23.50 | 7.53 | 17.40 | 17.72 | 22.12 | 22.73 | 22.29 | 20.01 |
